# Supplementary material for: Ceramides bind VDAC2 to trigger mitochondrial apoptosis
Source: Nat Commun. 2019 Apr 23;10:1832. doi: 10.1038/s41467-019-09654-4 (PMC6478893; doi:10.1038/s41467-019-09654-4)

SUPPLEMENTARY INFORMATION

Ceramides bind VDAC2 to trigger mitochondrial apoptosis

Shashank Dadsena, Svenja Bockelmann, John G. M. Mina, Dina G. Hassan, Sergei Korneev, Guilherme Razzera, Helene Jahn, Patrick Niekamp, Dagmar Müller, Markus Schneider, Fikadu G. Tafesse, Siewert J. Marrink, Manuel Nuno Melo, Joost C. M. Holthuis

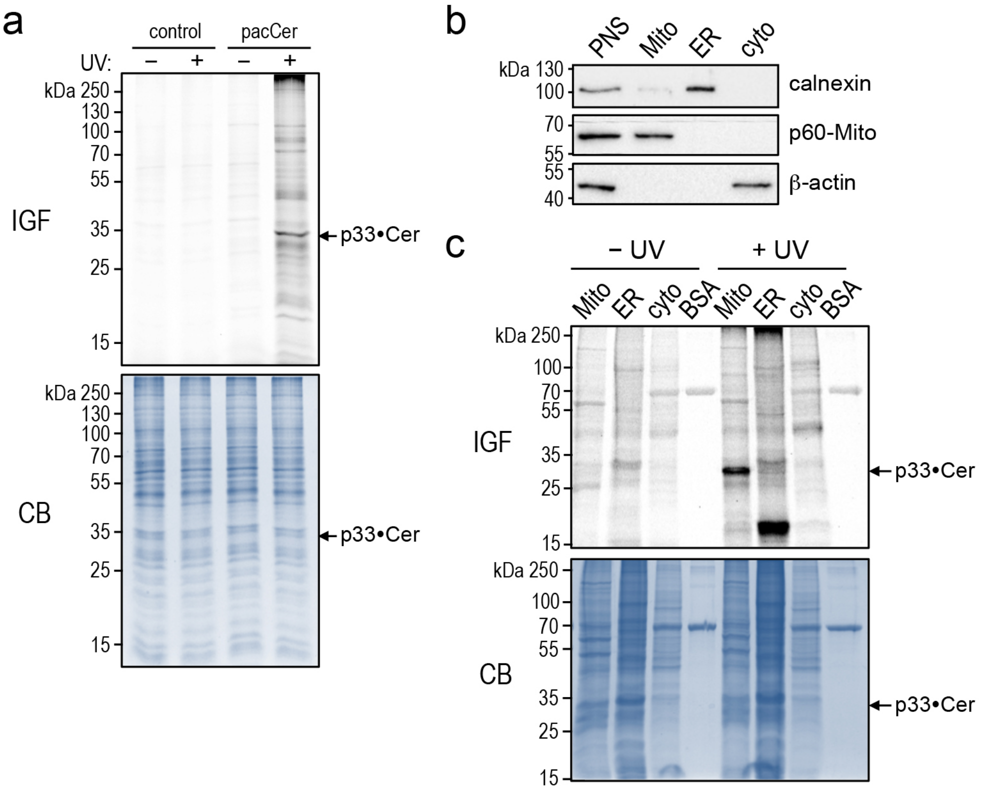

Supplementary Figure 1 | Mitochondria harbor a major candidate ceramide binding protein of 33kDa.

(**a**) Total HeLa cell membranes were incubated with control liposomes or liposomes containing 1 mol% pacCer for 1h at 37°C, UV-irradiated, and click-reacted with AF647-N_3. Samples were processed for SDS-PAGE, subjected to in-gel fluorescence (IGF) and stained with Coomassie blue (CB). Note that a membrane-bound protein band of ~33kDa, p33•Cer, was prominently and consistently photolabeled.

(**b**) A postnuclear supernatant (PNS) of lysed HeLa cells was subjected to subcellular fractionation, yielding cytosol (cyto) and membrane fractions enriched for mitochondria (Mito) or ER. Similar amounts of protein from each fraction were analyzed by immunoblotting against markers for ER (calnexin), mitochondria (p60-Mito) and cytosol (β-actin).

(**c**) Subcellular fractions from (b) were photolabeled with pacCer and processed as in (a), indicating that p33•Cer is a mitochondria-associated ceramide-binding protein.

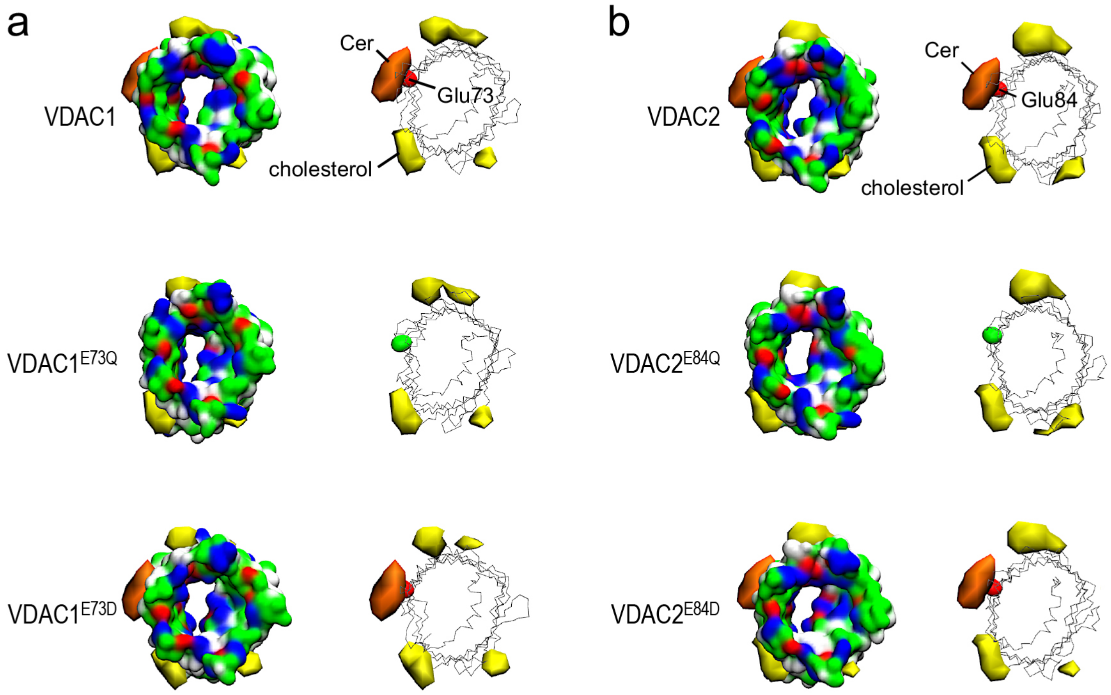

Supplementary Figure 2 | VDACs with a Glu-to-Asp substitution retain the ability to bind ceramide.

Space-filling and wireframe models of VDAC1, VDAC1^{E73Q}, VDAC1^{E73D}, VDAC2, VDAC2^{E84Q} and VDAC2^{E84D} with bilayer-facing deprotonated Glu and Asp residues. Indicated are the volumes for which there is ceramide occupancy greater than 10% (*orange*) or cholesterol occupancy greater than 20% (*yellow*).

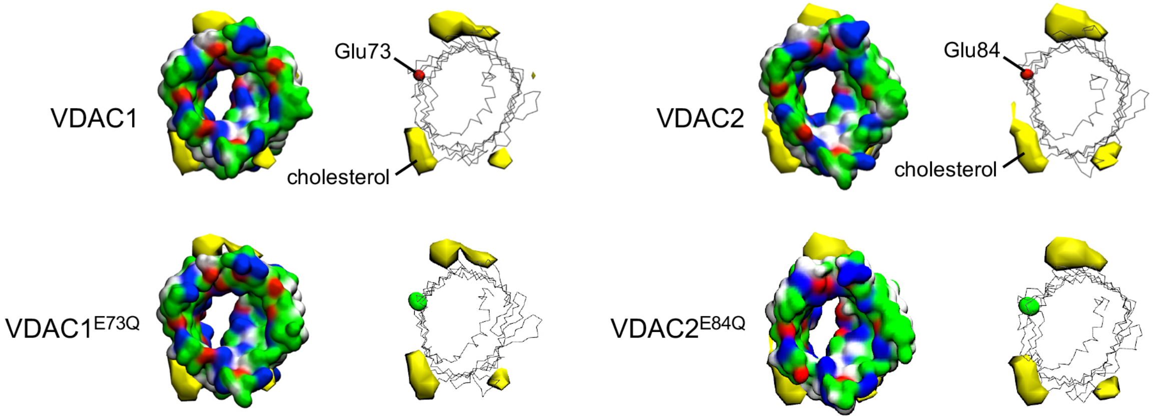

Supplementary Figure 3 | The binding sites for cholesterol and ceramide on VDAC channels are non-overlapping.

Space-filling and wireframe models of VDAC1, VDAC1^{E73Q}, VDAC2 and VDAC2^{E84Q}. Indicated are the volumes for which there is cholesterol occupancy greater than 20% (*yellow*) in MD simulations performed in the absence of ceramide. The position of the bilayer-facing deprotonated Glu residue involved in ceramide binding is marked. Note that cholesterol does not bind near the membrane-facing Glu also when ceramide is lacking, excluding the possibility that ceramide outcompetes cholesterol for its own binding site. Protein surface colours mark polar (*green*), apolar (*white*), cationic (*blue*) or anionic (*red*) residues.

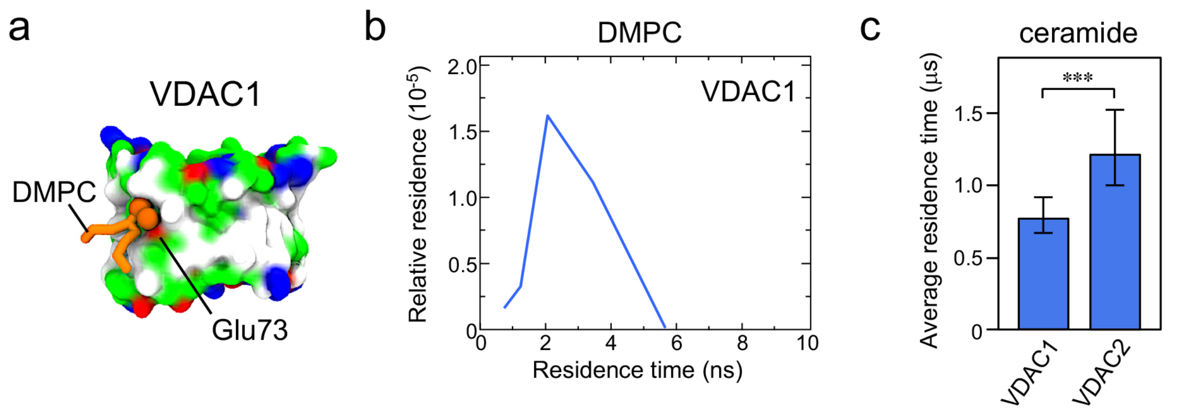

Supplementary Figure 4 | Average residence times of DMPC and ceramide bound to VDACs in MD simulations.

(**a**) Still from a MD simulation of mouse VDAC1 in a bilayer of 100% dimyristoyl-phosphatidylcholine (DMPC) showing binding of the phosphocholine head group of a DMPC molecule to the preferred ceramide binding site. The position of the bilayer-facing deprotonated Glu73 residue is indicated.

(**b**) Distribution of the durations of DMPC contacts at the preferred ceramide binding site. The *y*-axis indicates the fraction of the total system time spent in binding events of the duration indicated by *x*. Summing all points' *y*-values yields the fraction of total simulation time when DMPC was bound.

(**c**) Average residence times of ceramide at its preferred binding site were calculated from 1311 (VDAC1) and 710 binding-unbinding events (VDAC2) over an aggregated simulation time of 298 µs per protein. Error bars indicate 95% confidence intervals. ***$p < 0.005$ bytwo-tailed paired t-test.

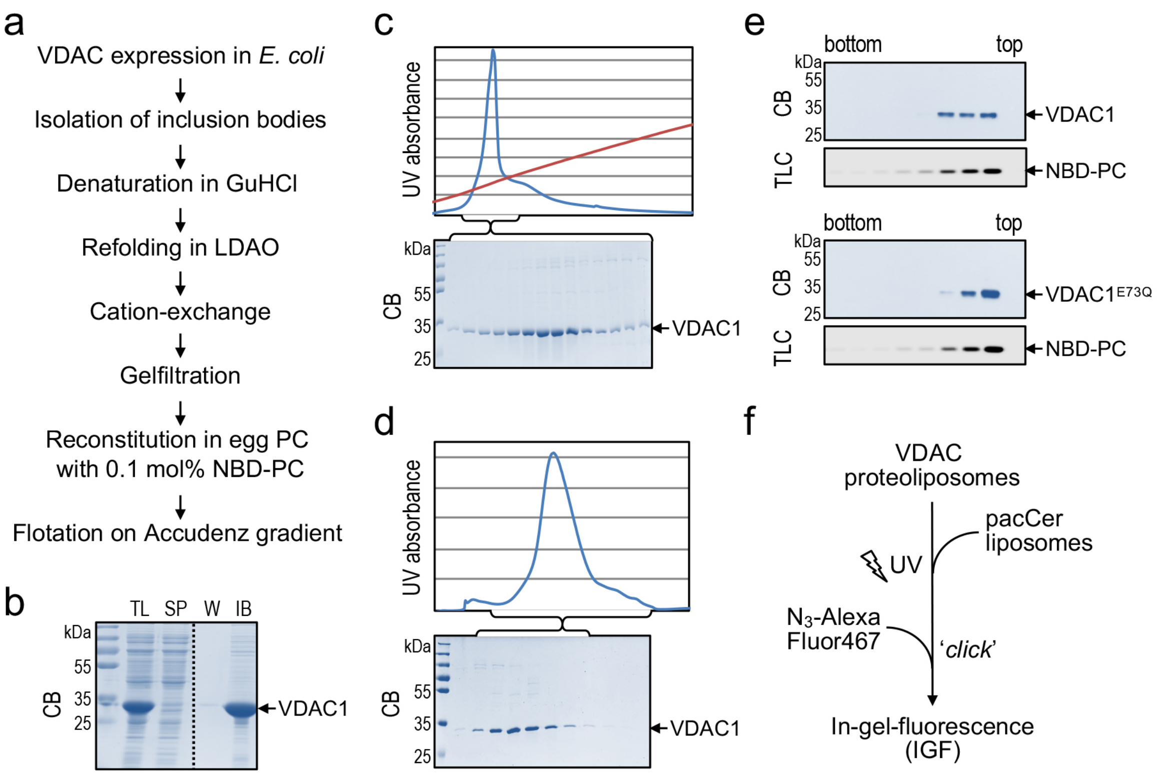

Supplementary Figure 5 | Purification and reconstitution of human VDAC channels produced in *E. coli*.

(**a**) Flow diagram outlining the purification and reconstitution of recombinant human VDAC proteins from *E. coli*.

(**b**) Inclusion bodies (IB) isolated from a total lysate (TL) of *E. coli* cells expressing human VDAC1 were analyzed by SDS-PAGE and Coomassie brilliant blue staining (CB). SP, supernatant; W, wash.

(**c**) Proteins in inclusion bodies isolated as in (b) were denatured in GuHCl, refolded in LDAO and subjected to cation-exchange column chromatography. Column fractions were analyzed by UV absorbance, SDS-PAGE and CB staining.

(**d**) Protein-peak fractions from (c) were subjected to gel-filtration column chromatography. Column fractions were analyzed by UV absorbance, SDS-PAGE and CB staining.

(**e**) Protein-peak fractions from (d) were reconstituted in egg PC spiked with 0.1 mol% NBD-PC. Reconstituted proteins were subjected to density gradient fractionation. Gradient fractions were analyzed by SDS-PAGE and CB staining or by TLC and NBD-fluorescence. Co-fractionation of VDAC1 and VDAC1^{E73Q} with NBD-PC are shown.

(**f**) Flow diagram outlining the photolabeling and IFG analysis of VDAC proteoliposomes.

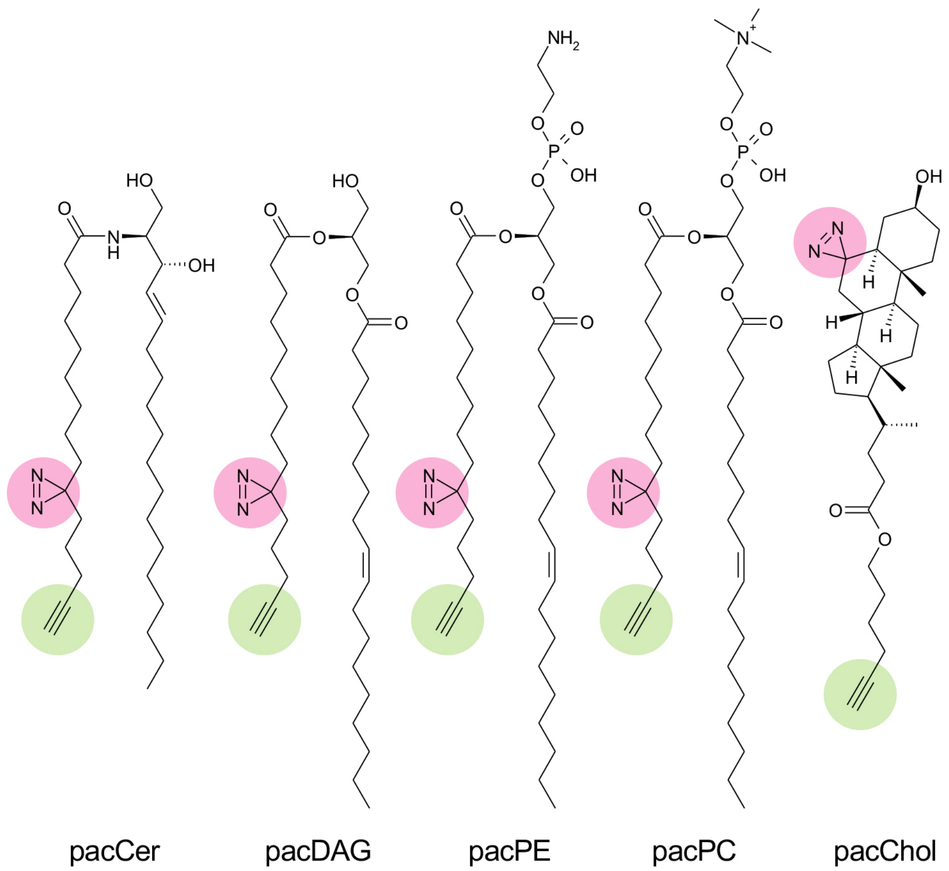

Supplementary Figure 6 | Structures of photoactive and clickable lipid analogues.
Structures of photoactive and clickable analogues of ceramide (pacCer), diacylglycerol (pacDAG), phosphatidylethanolamine (pacPE), phosphatidylcholine (pacPC) and cholesterol (pacChol). The clickable alkyne and photoactive diazirine groups are marked in green and violet, respectively.

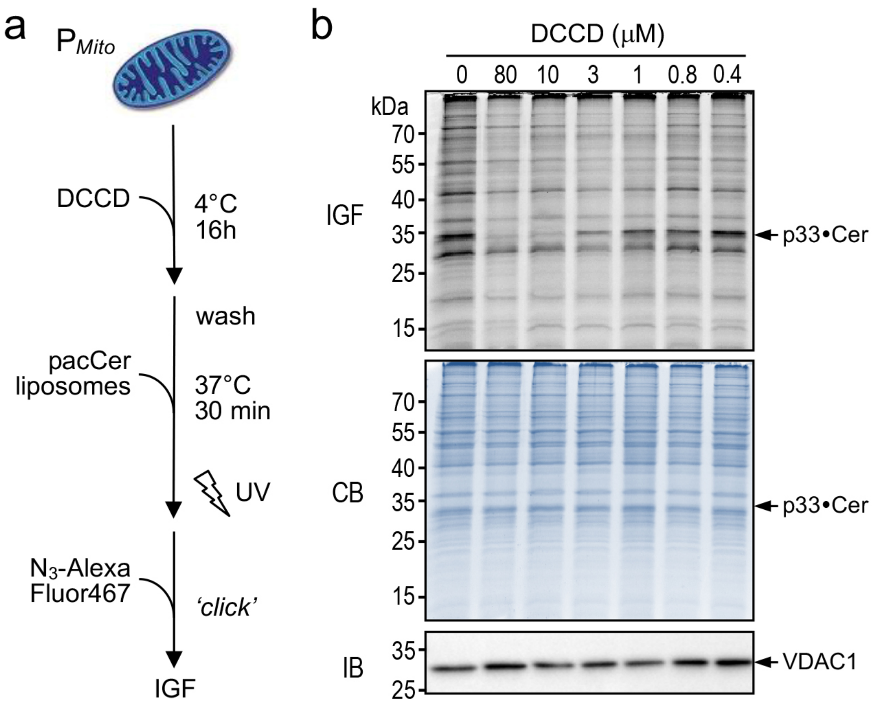

Supplementary Figure 7 | DCCD treatment of mitochondria abolishes pacCer labeling of native VDACs.

(**a**) Schematic outline of experimental procedure to determine the impact of DCCD on pacCer labeling of native VDAC channels in mitochondrial fractions isolated from HeLa cells.

(**b**) Crude mitochondrial fractions were pretreated with the indicated concentration of DCCD, washed, photolabeled with pacCer and then click-reacted with AF647-N_3. Next, fractions were subjected to SDS-PAGE and analyzed by IGF and CB staining or by immunoblotting (IB) using an antibody against VDAC1.

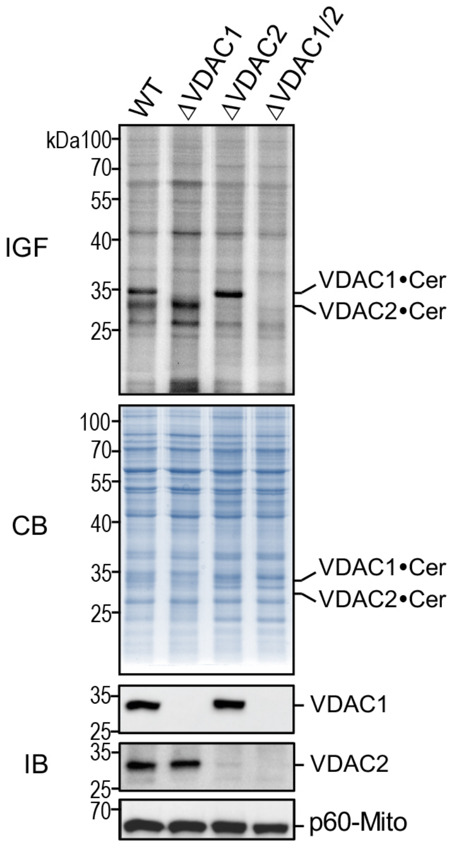

Supplementary Figure 8 | Genetic ablation of VDAC1 and VDAC2 results in loss of pacCer-labeled 33kDa-protein.

Human HCT116 colon cancer cell lines lacking VDAC1, VDAC2 or both were created by CRISPR/Cas9. Loss of VDAC1 and VDAC2 was confirmed by immunoblot (IB) analysis of mitochondrial pellets using antibodies against VDAC1, VDAC2 and mitochondrial marker protein p60-Mito. In addition, mitochondrial pellets were photolabeled with pacCer, click-reacted with AF647-N_3, subjected to SDS-PAGE and analyzed by IGF and CB staining. Note that removal of both VDAC1 and VDAC2 was accompanied by a complete loss of ~33kDa photolabeled protein bands.

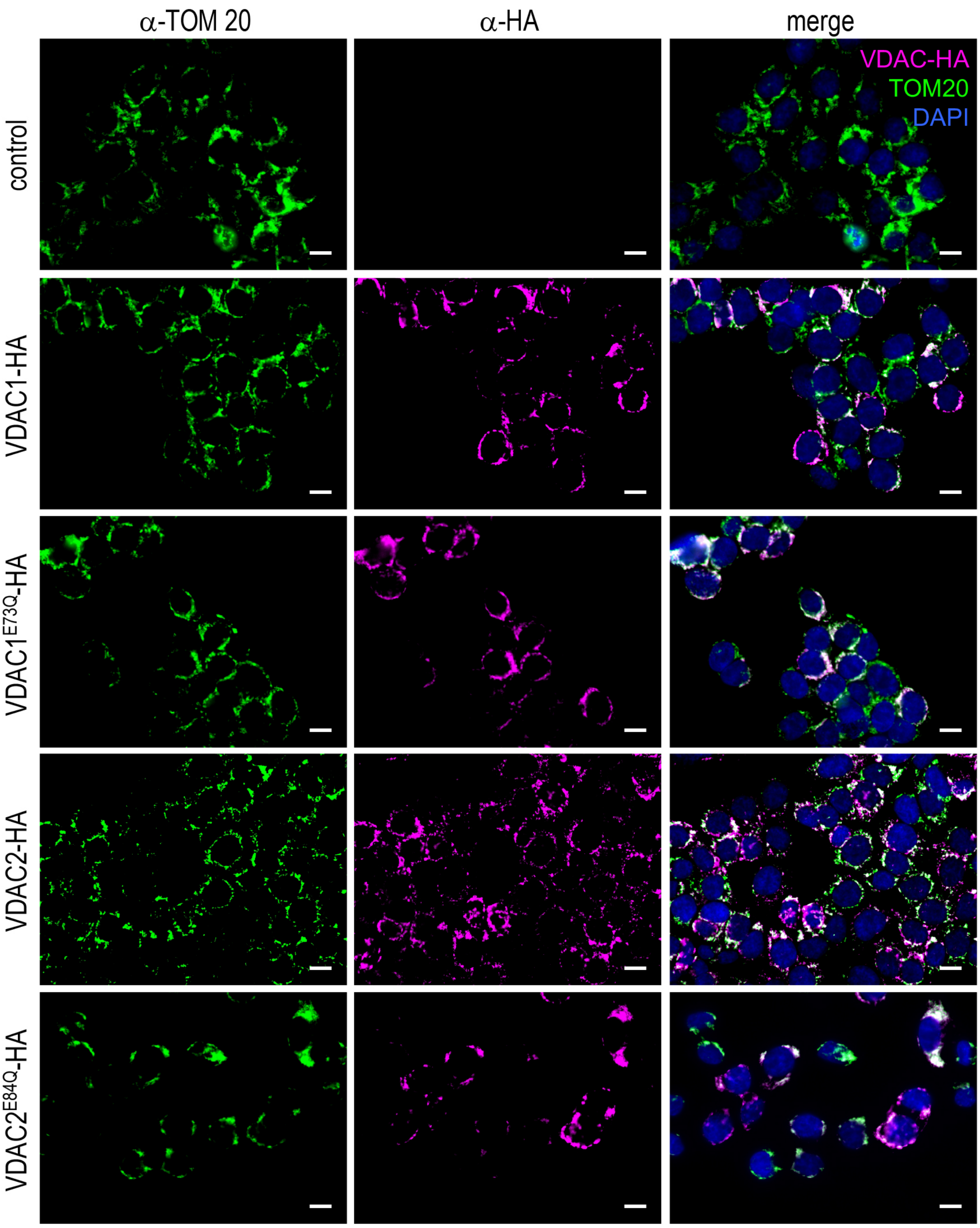

Supplementary Figure 9 | Immunofluorescence microscopy of HCT116 VDAC1/2 double KO cells transduced with HA-tagged VDAC proteins.

Human HCT116 VDAC1/2 double KO cells were stably transduced with HA-tagged VDAC1, VDAC1^{E73Q}, VDAC2 or VDAC2^{E84Q}. Next, cells were chemically fixed and then co-stained with DAPI (*blue*) and antibodies against the HA-tag (*red*) and the mitochondrial marker TOM20 (*green*). Scale bar, 10µm.

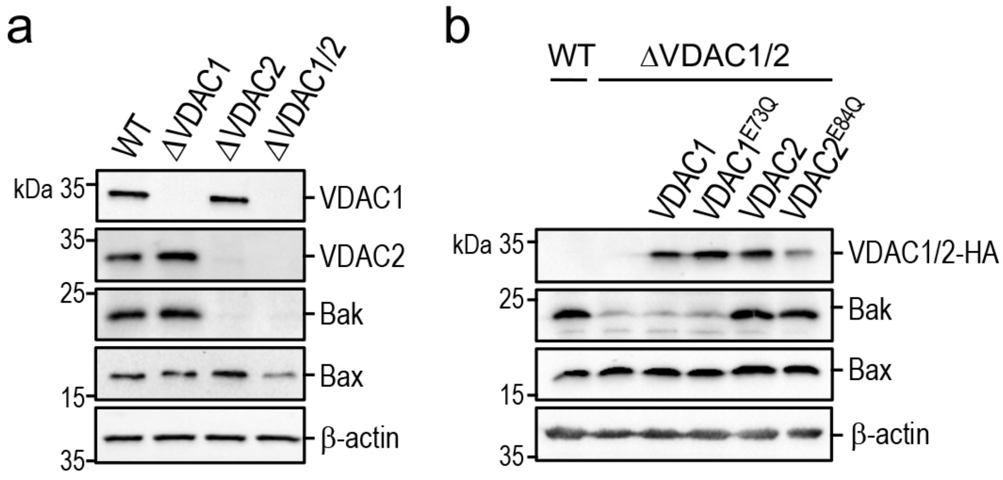

Supplementary Figure 10 | Membrane-facing Glu84 in VDAC2 is dispensable for stabilizing Bak.

(**a**) Wild-type (WT), VDAC1-KO (ΔVDAC1), VDAC2-KO (ΔVDAC1) and VDAC1/2-double KO (ΔVDAC1/2) HCT116 cells were processed for immunoblotting with antibodies against VDAC1, VDAC2, Bak, Bax and β-actin.

(**b**) Human HCT116 VDAC1/2 double KO cells were stably transduced with HA-tagged VDAC1, VDAC1^{E73Q}, VDAC2 or VDAC2^{E84Q} and then processed for immunoblotting with antibodies against the HA-epitope, Bak, Bax and β-actin.

Supplementary Table 1 | MS analysis of pacCer-photolabeled mitochondrial protein p33.

#	Accession #	Name	kDa	control #1	pacCer #1	control #2	pacCer #2
1	VDAC1_human	Voltage-dependent anion channel protein 1	30.8	0	5	0	3
2	VDAC2_human	Voltage-dependent anion channel protein 2	31.5	0	5	0	3
3	G3P_human	Glyceraldehyde-3-phosphate dehydrogenase	36.0	0	2	3	0
4	ACTB_human	Actin	41.7	0	0	0	2

Displayed are data from two independent experiments showing spectral counts for pacCer-labeled mitochondrial protein p33, which was affinity-purified from mitochondria that were UV-irradiated in the presence of pacCer-containing liposomes. Experiments in which pacCer was omitted from the liposomes served as control. Note that one of the spectral counts in the pacCer #1 and pacCer #2 experiments refers to a VDAC-derived peptide found in both VDAC1 and VDAC2 protein sequences.

Supplementary Table 2 | Simulated times per system.

System	Replica simulation times (μs)	Total simulation time (μs)
VDAC1	56, 28, 28, 28, 79, 79	298
VDAC2	54, 29, 29, 29, 78, 79	298
VDAC3	30	30
VDAC1^{E73Q}	56, 28, 28, 48	160
VDAC2^{E84Q}	24	24
VDAC1^{E73D}	56, 23, 22, 22	123
VDAC2^{E84D}	18	18
VDAC1 protonated	29, 23, 44	96
VDAC2 protonated	15, 17, 17	49
VDAC1 without ceramide	26	26
VDAC2 without ceramide	23	23
VDAC1^{E73Q} without ceramide	29	29
VDAC2^{E84Q} without ceramide	29	29
VDAC1 in 100% DMPC	27	27

Displayed are the simulated times for each system, discriminated for each replica. Unless stated otherwise, proteins were simulated in an OMM model supplemented with 5 mol% ceramide (see Methods). The main (VDAC1 and VDAC2) systems were simulated for a total of 0.6 ms (0.3 ms each). The grand total simulation time was 1.23 ms.

Supplementary Table 3 | Primers used in this study.

**Primer name**	**Primer sequence (5′–3′)**
pColdI-hVDAC1 F	ATCATATCGAAGGTAGGCACGCTGTGCCACCCAC
pColdI-hVDAC1 R	GCTTTTAAGCAGAGATTACCTATTTATGCTTGAAATTCCAGTCCTAGACCAAG
pColdI-hVDAC2 F	TGGAGCTCGGTACCCTCGAGATGGCGACCCACGGAC
pColdI-hVDAC2 R	GCTTTTAAGCAGAGATTACCTATCTAGATTAAGCCTCCAACTCCAGGGC
hVDAC1-E73Q F	ATGGACTGAGTACGGCCTGACGTTTACACAGAAATGG AATACCGAC
hVDAC1-E73Q R	GTCGGTATTCCATTTCTGTGTAAACGTCAGGCC
hVDAC2-E84Q F	GGTCTGACTTTCACACAAAAGTGGAACACTGATAAC
hVDAC2-E84Q R	GTTATCAGTGTTCCACTTTTGTGTGAAAGTCAGACC
pLNCX2-hVDAC1 F	TCATCACTCGAGGCAATGGCTGTGCCACCC
pLNCX2-hVDAC2 F	TCATCACTCGAGGCAATGGCGACCCACGGA
pLNCX2-hVDAC1/2 R	TCATACGCGGCCGCTCAGGCGTAATCCGGCACATCATAG

Uncropped Figures

Fig. 1e

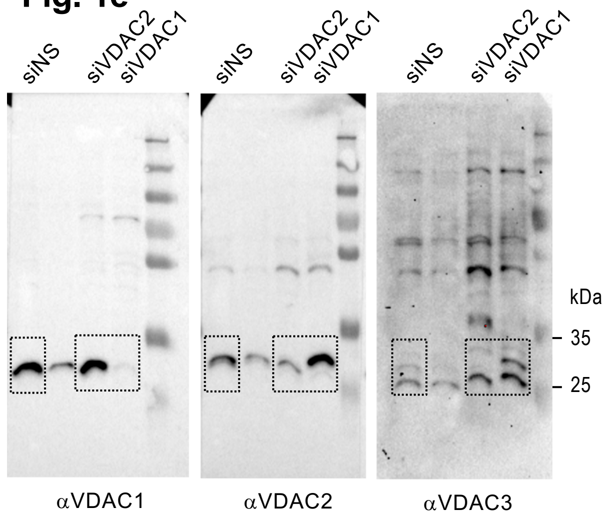

Fig. 1f

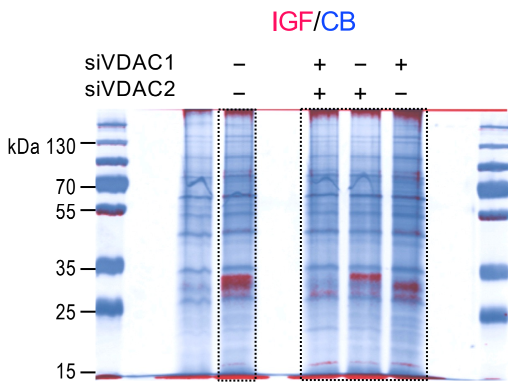

Fig. 1h

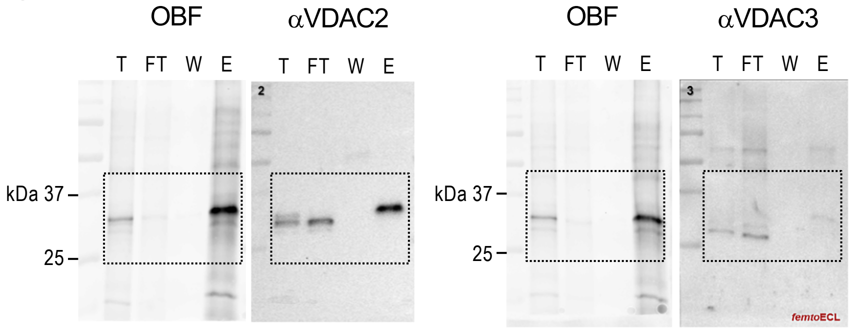

Fig. 3c

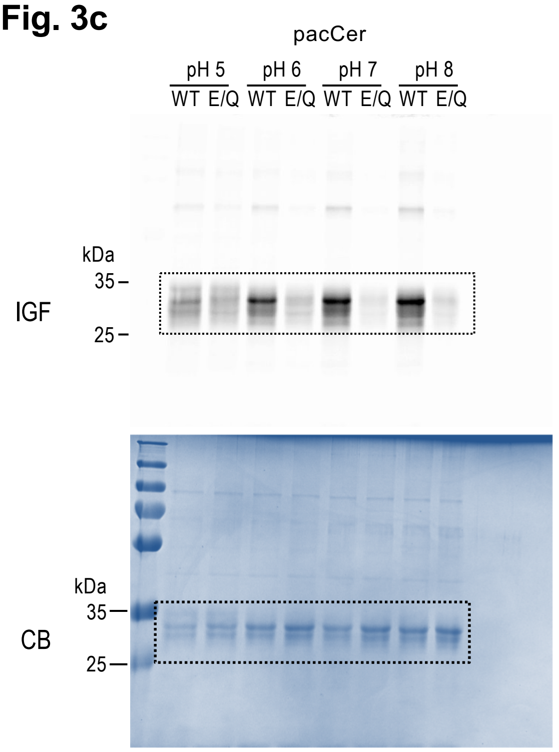

Fig. 5a

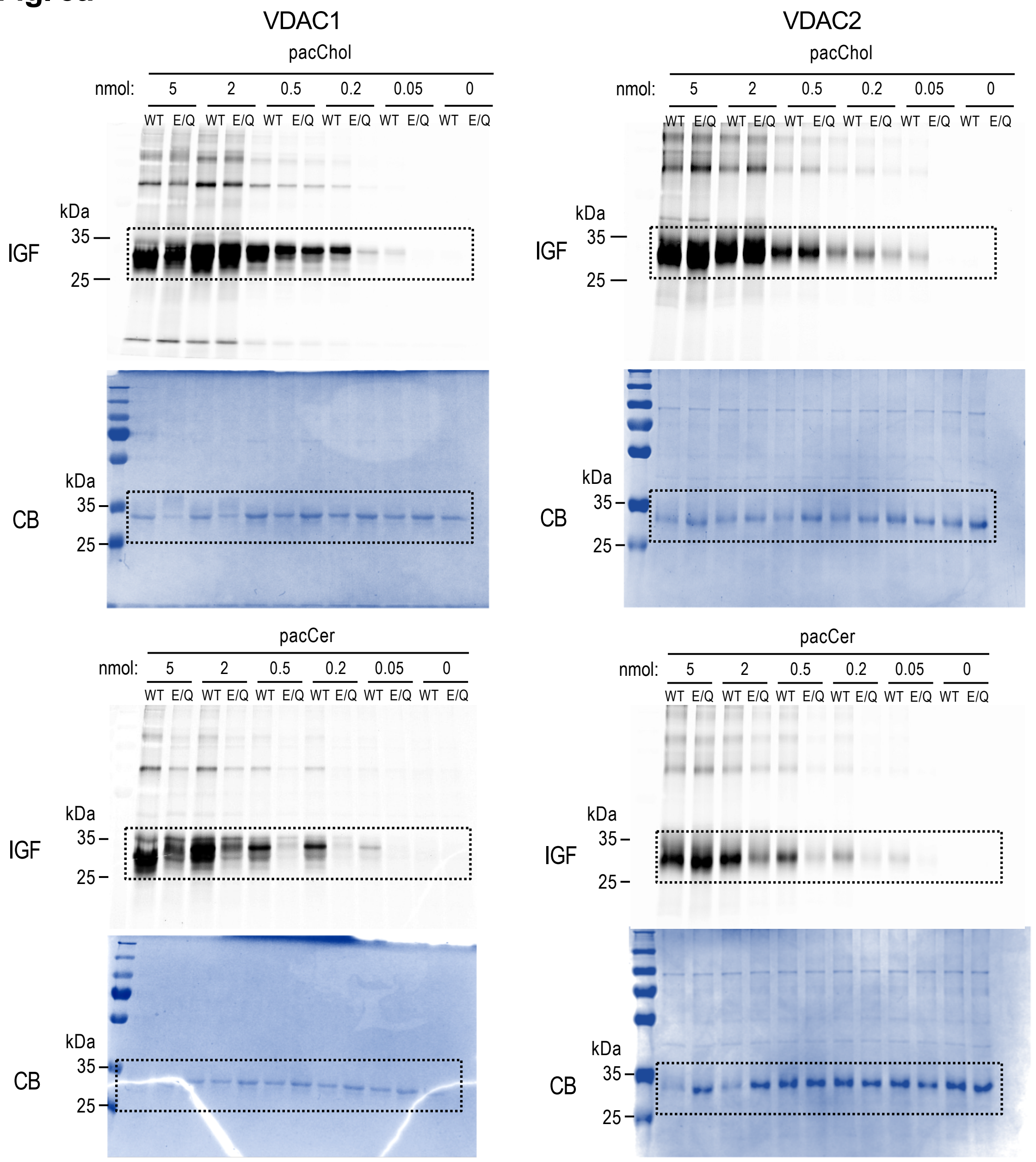

VDAC1
VDAC2
pacChol
nmol: 5 2 0.5 0.2 0.05 0
WT E/Q WT E/Q WT E/Q WT E/Q WT E/Q WT E/Q
kDa
35
25
IGF
CB
pacCer

Fig. 5b

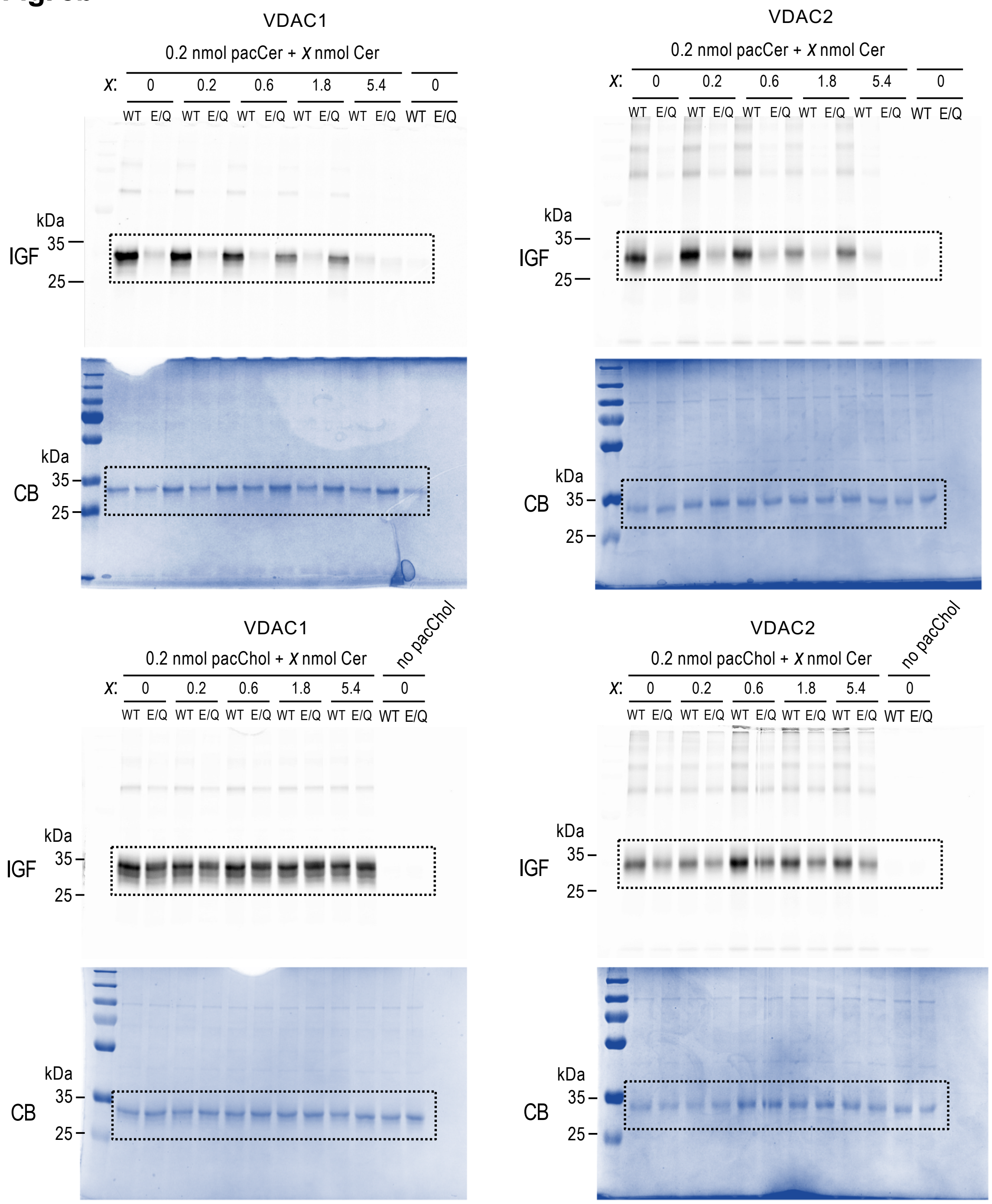
VDAC1
0.2 nmol pacCer + X nmol Cer
X: 0 0.2 0.6 1.8 5.4 0
WT E/Q WT E/Q WT E/Q WT E/Q WT E/Q WT E/Q
kDa
IGF
35
25
CB
VDAC2
0.2 nmol pacCer + X nmol Cer
VDAC1
0.2 nmol pacChol + X nmol Cer
no pacChol
VDAC2
0.2 nmol pacChol + X nmol Cer
no pacChol

Fig. 6c

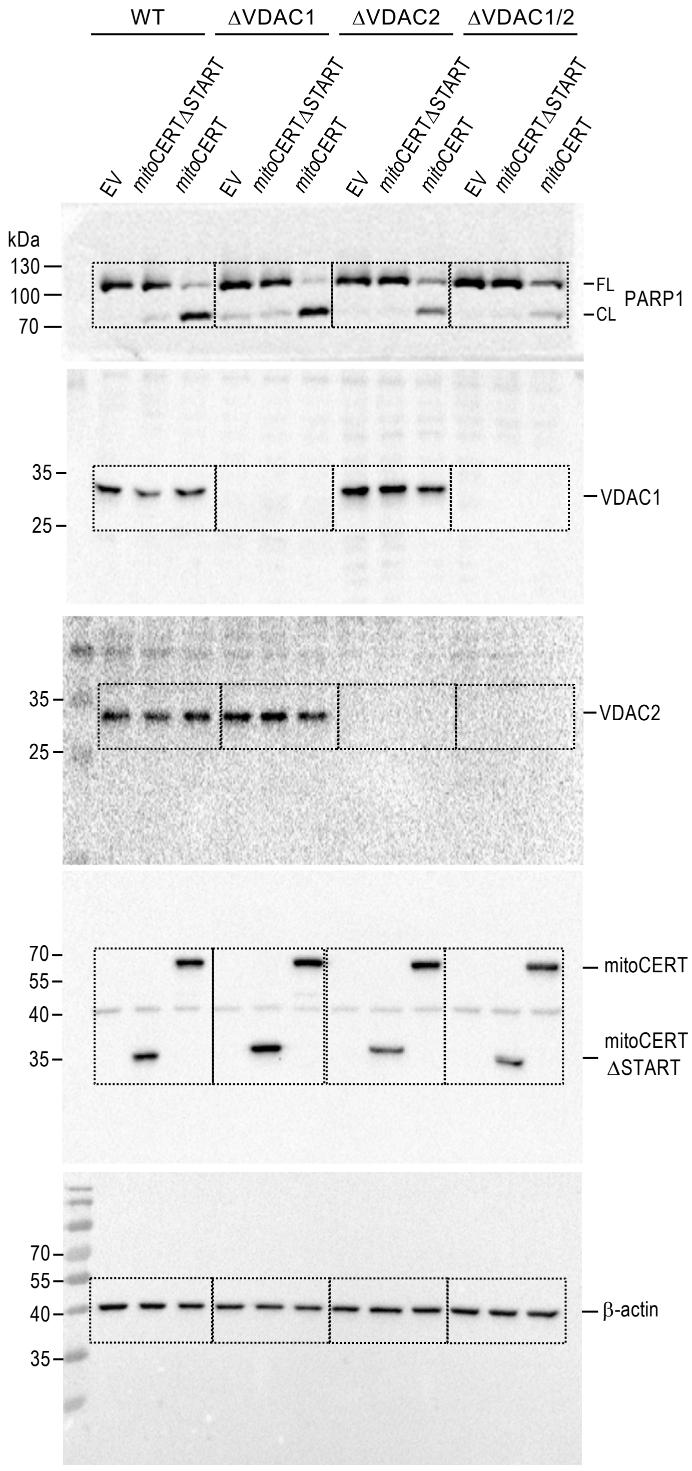
WT
ΔVDAC1
ΔVDAC2
ΔVDAC1/2
EV
mitoCERTΔSTART
mitoCERT
kDa
130
100
70
FL
CL
PARP1
35
25
VDAC1
VDAC2
70
55
40
35
mitoCERT
mitoCERT ΔSTART
β-actin

Fig. 7a

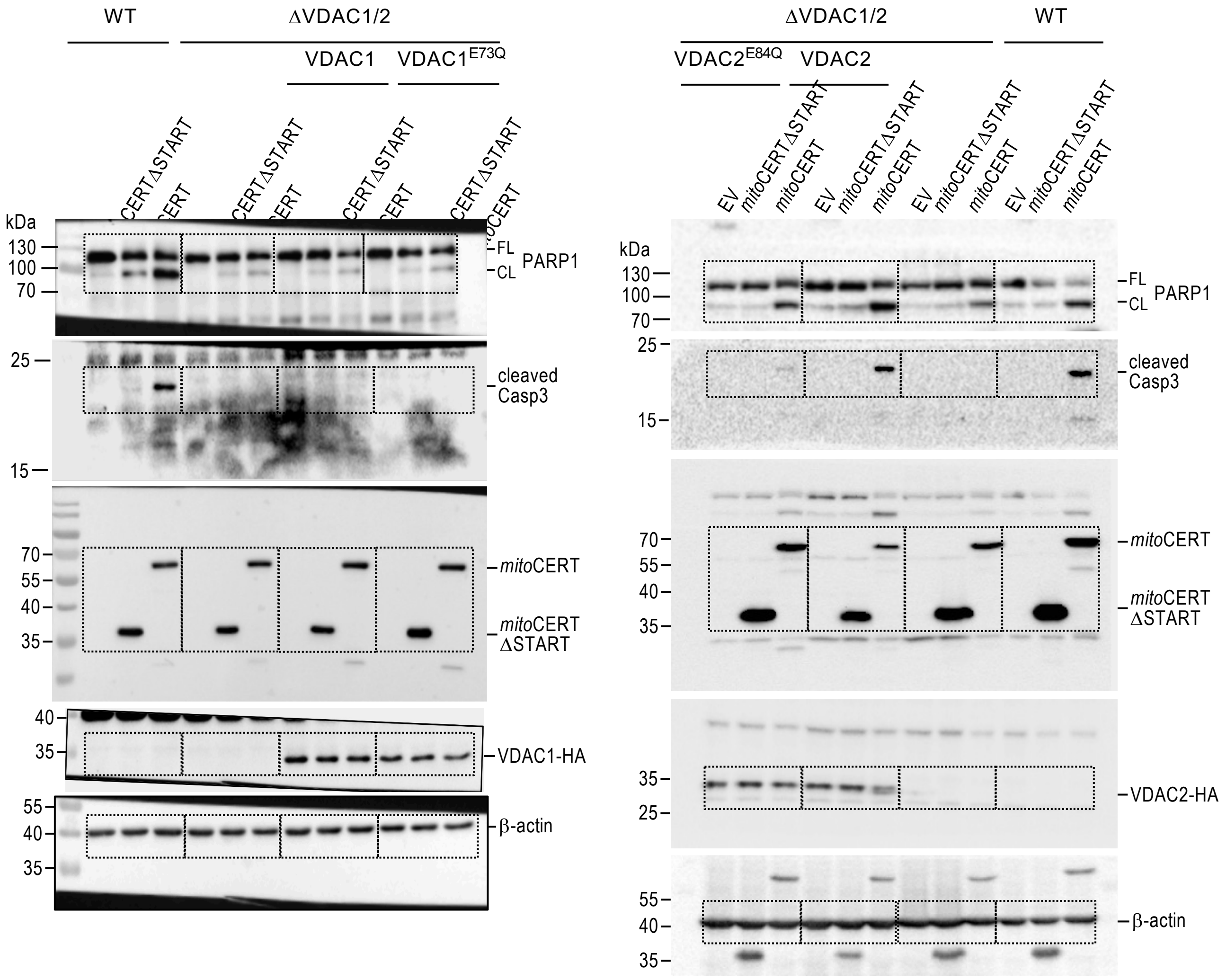

WT
ΔVDAC1/2
VDAC1
VDAC1E73Q
kDa
130
100
70
FL
CL
PARP1
25
15
cleaved
Casp3
70
55
40
35
mitoCERT
mitoCERT
ΔSTART
40
35
VDAC1-HA
55
40
35
β-actin
ΔVDAC1/2
WT
VDAC2E84Q
VDAC2
EV
mitoCERTΔSTART
mitoCERT
EV
mitoCERTΔSTART
mitoCERT
EV
mitoCERTΔSTART
mitoCERT
EV
mitoCERTΔSTART
mitoCERT
kDa
130
100
70
FL
CL
PARP1
25
15
cleaved
Casp3
70
55
40
35
mitoCERT
mitoCERT
ΔSTART
35
25
VDAC2-HA
55
40
35
β-actin

Suppl. Fig. 1b

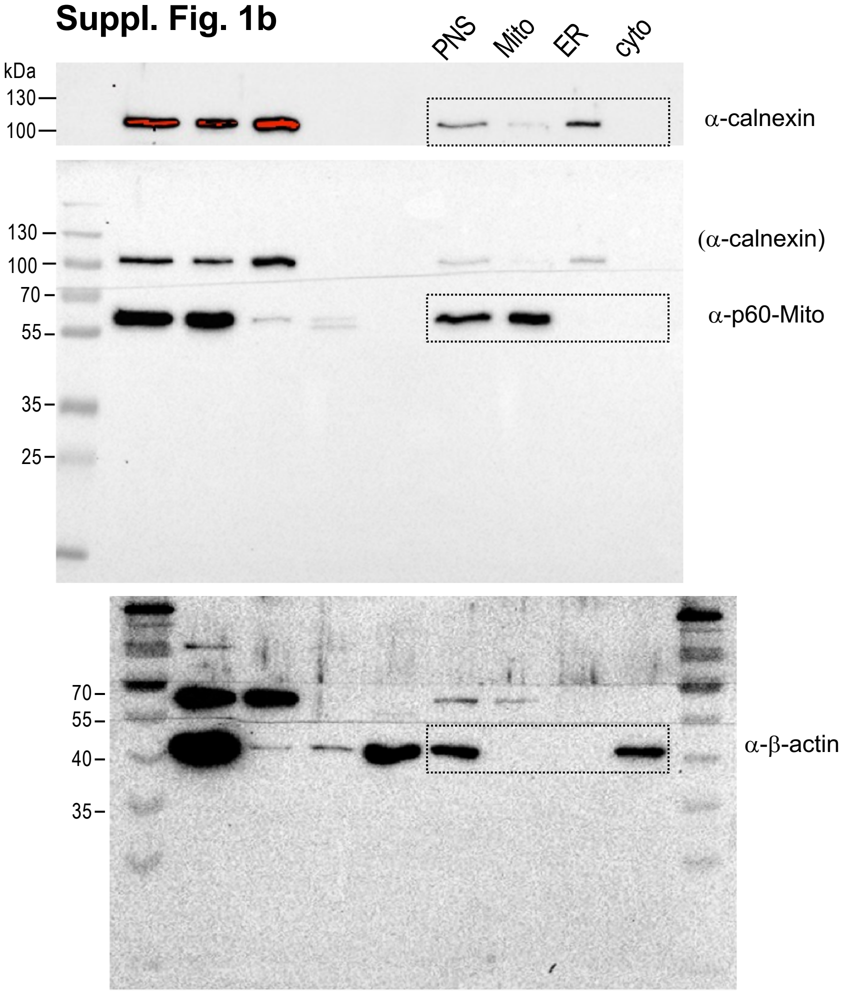

Suppl. Fig. 7b

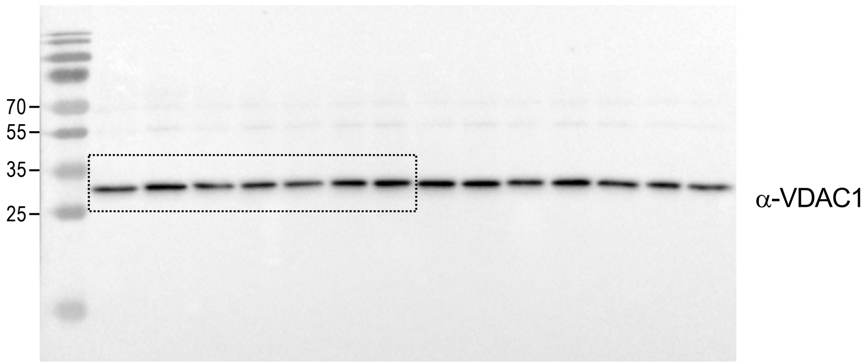

Suppl. Fig. 8

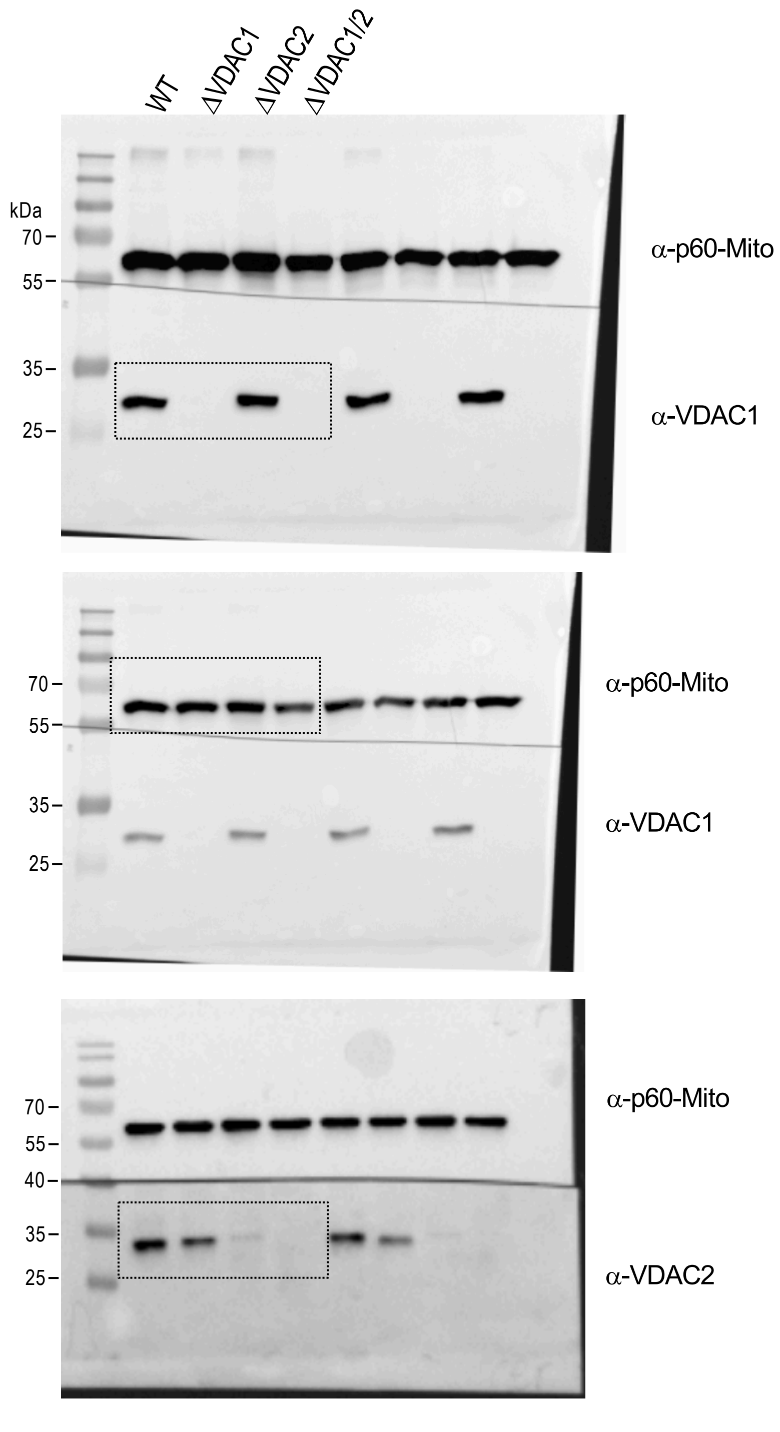
WT
ΔVDAC1
ΔVDAC2
ΔVDAC1/2
kDa
70
55
35
25
α-p60-Mito
α-VDAC1
70
55
35
25
α-p60-Mito
α-VDAC1
70
55
40
35
25
α-p60-Mito
α-VDAC2

Suppl. Fig. 10a

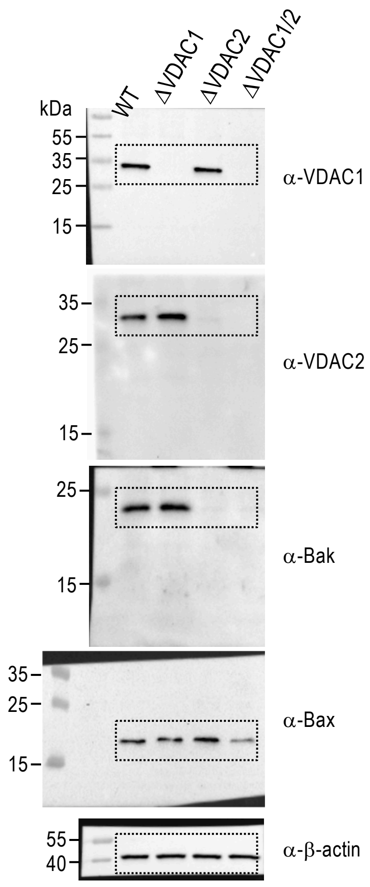

Suppl. Fig. 10b

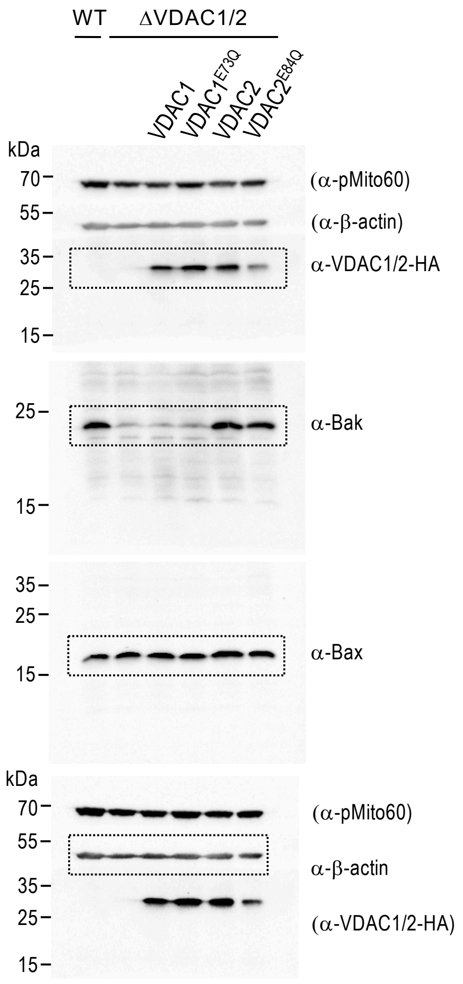

Supplement: Supplementary file 1 — Supplementary Information [file 41467_2019_9654_MOESM1_ESM.pdf]
