# Supplementary material for: Ceramides bind VDAC2 to trigger mitochondrial apoptosis
Source: Nat Commun. 2019 Apr 23;10:1832. doi: 10.1038/s41467-019-09654-4 (PMC6478893; doi:10.1038/s41467-019-09654-4)
Supplement: Supplementary file 4 — Description of Additional Supplementary Files [file 41467_2019_9654_MOESM4_ESM.pdf]

Title: Supplementary Movie 1 MD simulation of VDAC1 in an outer mitochondrial membrane model containing 5 mol% ceramide.
Description: MD simulation showing the approach and binding of a ceramide molecule on VDAC1 to the vicinity of membrane-facing glutamate residue Glu73 over a 2.3 µs period. Protein surface colours mark polar (green), apolar (white), cationic (blue) or anionic (red) residues.

Title: Supplementary Movie MD simulation of VDAC2 in an outer mitochondrial membrane model containing 5 mol% ceramide.
Description: MD simulation showing the approach and binding of a ceramide molecule on VDAC2 to the vicinity of membrane-facing glutamate residue Glu84 over a 6.0 µs period. Protein surface colours mark polar (green), apolar (white), cationic (blue) or anionic (red) residues.
