# Supplementary material for: Ceramides bind VDAC2 to trigger mitochondrial apoptosis
Source: Nat Commun. 2019 Apr 23;10:1832. doi: 10.1038/s41467-019-09654-4 (PMC6478893; doi:10.1038/s41467-019-09654-4)
Supplement: Supplementary file 5 — Reporting Summary [file 41467_2019_9654_MOESM5_ESM.pdf]

nature research

Corresponding author(s): Joost C. M. Holthuis

Reporting Summary

Nature Research wishes to improve the reproducibility of the work that we publish. This form provides structure for consistency and transparency in reporting. For further information on Nature Research policies, see Authors & Referees and the Editorial Policy Checklist.

Statistical parameters

When statistical analyses are reported, confirm that the following items are present in the relevant location (e.g. figure legend, table legend, main text, or Methods section).

n/a	Confirmed	
☐	☒	The exact sample size (*n*) for each experimental group/condition, given as a discrete number and unit of measurement
☐	☒	An indication of whether measurements were taken from distinct samples or whether the same sample was measured repeatedly
☐	☒	The statistical test(s) used AND whether they are one- or two-sided<br>*Only common tests should be described solely by name; describe more complex techniques in the Methods section.*
☒	☐	A description of all covariates tested
☒	☐	A description of any assumptions or corrections, such as tests of normality and adjustment for multiple comparisons
☐	☒	A full description of the statistics including central tendency (e.g. means) or other basic estimates (e.g. regression coefficient) AND variation (e.g. standard deviation) or associated estimates of uncertainty (e.g. confidence intervals)
☐	☒	For null hypothesis testing, the test statistic (e.g. F, t, r) with confidence intervals, effect sizes, degrees of freedom and P value noted<br>*Give P values as exact values whenever suitable.*
☒	☐	For Bayesian analysis, information on the choice of priors and Markov chain Monte Carlo settings
☒	☐	For hierarchical and complex designs, identification of the appropriate level for tests and full reporting of outcomes
☒	☐	Estimates of effect sizes (e.g. Cohen's d, Pearson's r), indicating how they were calculated
☐	☒	Clearly defined error bars<br>*State explicitly what error bars represent (e.g. SD, SE, CI)*

Our web collection on statistics for biologists may be useful.

Software and code

Policy information about availability of computer code

Data collection	Raw MS data were converted to peak lists using Data Analyzer 4.1 (Bruker, USA) and noise filtered using an in-house developed script (available upon request). The spectra were searched with Mascot (precursor mass tolerance 0.8 Da; product mass tolerance 0.4 Da; 2 missed cleavages) against all human proteins in the Swissprot (v56.2) database. Peptide identifications were accepted with a score greater than 20 and a p-value smaller than 0.01, and proteins were identified with at least two unique peptides. Structures of all three mouse VDAC isoforms were obtained using pyMOL software, using a well-resolved structure of mouse VDAC1 (PDB:4C69) as base. Simulations were run with GROMACS version 5.0.
Data analysis	In-gel fluorescence intensities were quantified using ImageQuant TL software. The amount of recombinant protein was determined by measuring Coomassie blue staining intensity against that of a reference protein (BSA) using Image Lab 5.2 software (Bio-Rad Laboratories). Analysis algorithms were programmed in Python, using MDAnalysis (https://www.mdanalysis.org) and NumPy packages. The MDreader package (https://github.com/mnmelo/MDreader) was used to allow analysis parallelization, which was essential in tackling the multi-gigabyte trajectory analysis in a short time. Visual molecular dynamics (VMD) software was used to create images and videos.

For manuscripts utilizing custom algorithms or software that are central to the research but not yet described in published literature, software must be made available to editors/reviewers upon request. We strongly encourage code deposition in a community repository (e.g. GitHub). See the Nature Research guidelines for submitting code & software for further information.

Data

Policy information about availability of data

All manuscripts must include a data availability statement. This statement should provide the following information, where applicable:
- Accession codes, unique identifiers, or web links for publicly available datasets
- A list of figures that have associated raw data
- A description of any restrictions on data availability

All relevant data are available from the corresponding authors on request.

Field-specific reporting

Please select the best fit for your research. If you are not sure, read the appropriate sections before making your selection.

☒ Life sciences ☐ Behavioural & social sciences ☐ Ecological, evolutionary & environmental sciences

For a reference copy of the document with all sections, see nature.com/authors/policies/ReportingSummary-flat.pdf

Life sciences study design

All studies must disclose on these points even when the disclosure is negative.

Sample size	Sample sizes were determined based on the author's experience of what is necessary to generate a convincing and compelling result.
Data exclusions	No data were excluded from the analyses in the experiments.
Replication	All experimental findings were reliably reproduced at least once, using independent experimental samples.
Randomization	N/A
Blinding	No blinding was done in this study. Virtually all the data are quantitative. Most measurements are made using a machine and not easily subject to operator bias.

Reporting for specific materials, systems and methods

Materials & experimental systems

n/a	Involved in the study	
☒	☐	Unique biological materials
☐	☒	Antibodies
☐	☒	Eukaryotic cell lines
☒	☐	Palaeontology
☒	☐	Animals and other organisms
☒	☐	Human research participants

Methods

n/a	Involved in the study	
☒	☐	ChIP-seq
☒	☐	Flow cytometry
☒	☐	MRI-based neuroimaging

Antibodies

Antibodies used	Monoclonal anti-β-actin (Sigma-Aldrich, A1978; IB 1:50,000), rabbit polyclonal anti-FLAG (Cell Signaling, 2368; IB 1:1,000), mouse monoclonal anti-mitochondrial surface protein p60 (Millipore, MAB1273; IB 1:1,000), rabbit polyclonal anti-VDAC1 (Cell Signaling, 4661; IB 1:1,000), goat polyclonal anti-VDAC2 (Abcam, Ab37985; IB 1:1,000), rabbit polyclonal anti-VDAC3 (Abcam, Ab80452; IB 1:1,000), mouse monoclonal anti-TOM20 (Millipore, Mabt166; IF 1:200;), rabbit polyclonal anti-TOM40 (Abcam, Ab185543; IB 1:1,000), rabbit polyclonal anti-HA (Invitrogen, 715500; IB 1:1,000; IF 1:200), rat monoclonal anti-HA (Roche, 12158167001; IB 1:1,000), rabbit monoclonal anti-Bax (Cell Signaling, 5023; IB 1:1,000), rabbit polyclonal anti-cleaved caspase-3 (Cell Signaling, 96611; IB 1:1,000), mouse monoclonal anti-PARP-1 (Santa Cruz, sc8007; IB 1:1,000) and rabbit polyclonal anti-calnexin (Santa Cruz , sc11397; IB 1:1,000).
Validation	Specificity of anti-VDAC antibodies was verified by immunoblot analysis of total lysates from siVDAC-treated HeLa cells or VDAC-KO HCT116 cells. All other commercial antibodies were validated by the suppliers.

Eukaryotic cell lines

Policy information about cell lines

Cell line source(s)	HEK293T cells, Thermo Scientific, cat. no. HCL4517; HeLa cells, ATTC, CCl-2; HCT116 cells, ATTC, CCL-247.
Authentication	Cell line authentication was not performed as cells were not listed in the commonly misidentified category.
Mycoplasma contamination	All cell-lines were mycoplasma-free.
Commonly misidentified lines (See ICLAC register)	No commonly misidentified cell lines were used.
